# Differential diagnosis between AML infiltration, lymphoma and tuberculosis in a patient presenting with fever and mediastinal lymphadenopathy: A case report

**DOI:** 10.3892/ol.2014.1785

**Published:** 2014-01-08

**Authors:** NA ZHAO, JUN-JIE YANG, GUANG-SEN ZHANG

**Affiliations:** 1 Division of Hematology, The Second Xiangya Hospital, Central South University, Changsha, Hunan 410011, P.R. China; 2 Division of Hematology, Yijishan Hospital, Wannan Medical College, Wuhu, Anhui 241001, P.R. China

**Keywords:** tuberculosis, lymphadenopathy, leukemia

## Abstract

The diagnosis of tuberculosis in immunocompromised hosts is often difficult as the hosts have atypical tuberculosis symptoms. The current study presents a case of scrofula and pulmonary tuberculosis with acute myelocytic leukemia (AML). As the disease became aggravated, the patient presented with fever, hemophagocytosis in the bone marrow, lymphadenopathy of the supraclavicular fossa, and mediastinal and nodular shadow in the chest by computed tomography. The symptoms presented successively or were coexistent, which made differentiation between tuberculosis, lymphoma, AML infiltration or other infections challenging. The diagnosis of tuberculosis was based on clinical and radiographic observations, morphological observation of the biopsies and the positive effect of antituberculosis drugs, while Ziehl-Neelsen stainings for acid fast bacilli were negative. The patient was treated with antituberculosis drugs, while receiving chemotherapy for AML. It is important to distinguish tuberculosis in adults with AML from other causes of fever, mediastinal masses in radiographic observations and hemophagocytosis in the bone marrow.

## Introduction

Infection is the leading cause of morbidity and mortality among patients with hematological malignancies (1). Previously, Gupta *et al* retrospectively studied six cases with tuberculosis among 382 acute lymphoblastic leukemia patients with febrile episodes; the rate of infection with tuberculosis was 1.57% (2). Patients with hematological malignancies have been reported to develop tuberculosis during or following chemotherapy (1,3). In addition, the diagnosis of tuberculosis is often difficult to determine due to its atypical symptoms, various radiographic observations and other examinations, such as negative purified protein derivative (PPD) skin test. Therefore, it is necessary to pay more attention to these patients.

The current study describes a case of scrofula and pulmonary tuberculosis with acute myelocytic leukemia (AML). Notably, the tuberculosis infection may have existed prior to the diagnosis of AML and then become aggravated during and following the chemotherapy. In addition, the patient presented with fever, lymphadenopathy and hemophagocytosis in the bone marrow. The current case report highlights that clinicians must be alerted to atypical characteristics of tuberculosis infection, particularly among patients with hematological malignancies.

## Case report

In November 2010, a 23-year-old male was referred to the Hematology Clinic of the Second Xiangya Hospital (Changsha, China) with a fever and dry cough for one week. The patient’s blood routine (BR) showed a white blood cell count of 76.8×10^9^/l, hemoglobin count of 6.6 g/dl, red blood cell count of 1.87×10^12^/l and platelet count of 31×10^9^/l, with 87.80% neutrophils. Subsequently, the patient was admitted to the Department of Hematology (The Second Xiangya Hospital, Central South University, Changsha) for further evaluation. The patient had no history of tuberculosis in the family.

On admission, the patient had a body temperature of 39.0°C. No abnormality was identified on physical examination. In the peripheral blood work, lactic dehydrogenase (LDH) was high at 937.5 μ/l (upper limit of normal, 245.0 μ/l) and bone marrow aspiration revealed 77% myeloblasts. Peroxidase staining was positive and immunophenotype examination revealed that these cells had originated from myeloblastic cell lines. In addition, AML1/ETO fusion gene expression was positive. X-ray chest radiograph showed enlargement of the left hilar, laminar shadow and marginal infiltration in the right lower lung lobe. Therefore, the diagnosis of AML-M2a was determined and the induction chemotherapy was initiated, with empiric antibiotics.

However, although the BR and bone marrow aspiration indicated that the patient had achieved remission 14 days following the first cycle of chemotherapy, the patient continued to complain of repeated irregular fever and occasional frothy sputum, which did not respond to the broad-spectrum antibiotic and antimycotic treatments. During that time, sputum acid-fast smear was negative three times, and blood and sputum cultures for bacteria and fungi were sterile five times.

**Figure 1:**
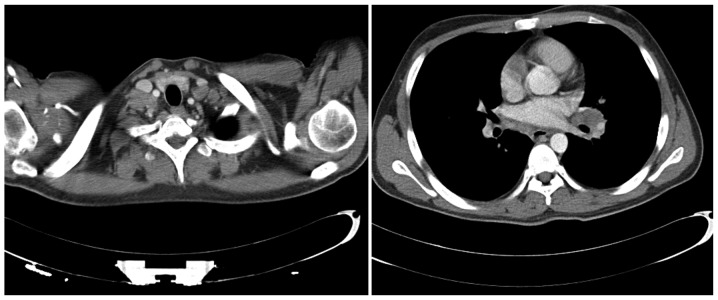
Contrast-enhanced image shows multiple enlarged lymph nodes in the bilateral supraclavicular fossa, mediastinum and left hilus.

One month following the completion of the induction chemotherapy, the patient was administered the second cycle of chemotherapy. In order to identify the origin of the fever, bone marrow aspiration was performed again, which presented the morphology of myeloblasts to be roughly normal, accounting for 3%, with occasional hemophagocytosis. Computed tomography (CT) of the brain, abdomen and pelvis showed splenomegaly; chest CT identified multiple enlarged lymph nodes in the bilateral supraclavicular fossa, mediastinum and left hilus and a number of lymph nodes that had become confluent masses, with a maximum size of 3×4 cm (Fig. 1). In addition, the blood C-reactive protein levels were 81.70 mg/l (upper limit of normal, 8.00 mg/l), ferritin levels were 2,098.27 ng/ml (upper limit of normal, 274.66 ng/ml), LDH levels were 382.5 μ/l and fibrinogen levels were 522 mg/dl (upper limit of normal, 400 mg/dl). The immunological examination for infections of bacteria, viruses and parasites suggested PPD-antibody (Ab) IgG (+), MycoDot™ (+), Widal reaction (−) and galactomannan (GM) test (−), and cardiac color ultrasound revealed marginal pericardial effusion. The patient exhibited 8 mm of induration in response to the PPD skin test. The patient was administered a diagnostic treatment, including isoniazid, rifampicin, pyrazinamide and fluoroquinolones. One week later, due to poor liver function, the rifampicin was terminated and the patient was discharged with prescriptions for isoniazid and pyrazinamide only, in January, 2011.

**Figure 2:**
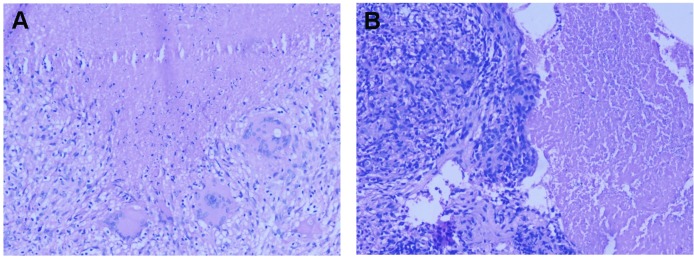
Histological view of biopsy specimens obtained from (A) surgical excision of the supraclavicular fossa lymph node and (B) fiberoptic bronchoscopy. The two images show caseous necrosis, epithelioid cell nodules and Langhans giant cell. Magnification, ×100.

One month later, the patient was referred to our department again for the second consolidation chemotherapy with no complaints of fever and cough. However, several peanut-sized lymph nodes were palpated in the right supraclavicular fossa and subclavicular area, without tenderness. CT revealed enlarged lymph nodes in the bilateral supraclavicular fossa, mediastinum and left hilus. Some of the nodes had merged into masses, the maximum diameter of which was 3 cm. The air tube and bronchus were marginally compressed and bilateral pleural thickening was observed. The patient then underwent surgical excision of the supraclavicular fossa lymph node. The histology of the specimen showed caseous necrosis, epithelioid cell nodules and multinuclear giant cells (Fig. 2A). However, the acid fast bacilli (AFB) staining of this specimen was negative. Due to the liver dysfunction (high levels of transaminase and total bilirubin), only two antituberculosis drugs (isoniazid and ethambutol) and the second consolidation chemotherapy were administered simultaneously. The surgical incision healed well and the patient was discharged with prescriptions for these two antituberculosis drugs and liver protective drugs.

During the ensuing two months, the patient frequently had a cough with no fever and the biopsy incision became infected, covered by a yellow-green pyogenic moss. BR, bone marrow aspiration and AML1-ETO fusion gene showed that the patient had achieved complete remission. In addition, chest CT revealed a few massive dot shadows in the left upper lobe, with obscured edges and evidently enlarged lymph nodes of the mediastinum and left hilus, with the neighboring bronchus compressed and narrowed. Due to the fact that the tuberculosis had been poorly controlled and lung deterioration was radiographically observed, chemotherapy was interrupted. The patient received three antituberculosis drugs (isoniazid, rifapentin and ethambutol). Two months following the initiation of the three antituberculosis drug treatments, the patient continued to occasionally present with a mild fever during the afternoon; however, the patient’s temperature returned to normal levels without special treatment. In addition, the dry cough continued and a yellow-green purulent ulcer continued to exist in the biopsy incision, the size of which was 1.0×0.5 cm. The patient exhibited 22 mm of induration in response to the PPD skin test and GM test (−), PPD-IgG Ab (+) and MycoDot™ (+). Chest CT showed a nodular shadow in the dorsal segment of right lower lung, with rough edges, thickening bilateral bronchovascular bundles and multiple enlarged lymph nodes in the mediastinum. CT-guided percutaneous needle lung biopsy (PNLB) and fiberoptic bronchoscopy (FB) were performed. PNLB was performed several times, but all attempts failed to reach the nodule, as shown by CT. The results of FB revealed that red granulomatous materials existed at the opening of the left main bronchus, where the submucosa was swollen and congested. In addition, the opening of the left lingular bronchus exhibited red granulomatous elements, without obstruction of the lumen. However, red granulomatous materials and white necrotic elements obstructed the opening when the biopsy was performed. These observations were consistent with the diagnosis of bronchial tuberculosis (lesions of the left main bronchus, left lingular lobe and left lower lobe) and scrofula (ruptured lumens). The pathology of the specimen showed abundant coagulative necrosis, epithelioid nodules and a few Langhans giant cells (Fig. 2B). The AFB staining of the biopsy specimen and lavage fluid were negative. However, considering the patient’s poor liver function and adverse reaction of liver damage by rifapentine, isoniazide and ethambutol, plus strong liver protective drugs were administered. Simultaneously, the fifth cycle of chemotherapy was initiated. During hospitalization, the patient’s cough improved. To date, the patient’s condition has remained stable and the AML is in remission. The patient was monitored for one year, without tuberculosis and leukemia relapse. The patient provided written informed consent.

## Discussion

Commonly, the symptoms of tuberculosis, which include fever, cough, lymphadenopathy and loss of appetite and weight, are atypical (1,4), making the diagnosis more difficult. Persistent pyrexia in patients with malignant hematological disorders, in remission, may serve as a diagnostic marker for tuberculosis (5). The fever of the present patient existed prior to the diagnosis of AML and continued from admission to the remission period. In addition, the empiric antibiotics appeared to be inefficient, even when AML was in remission, which led to the suspicion of other uncommon reasons, such as tuberculosis infection.

Long-lasting hematogenous, lymphogenous, endobronchial and local spread of the disease results in variable radiological appearances (6). Previously, Al-Anazi *et al* considered that the radiographic appearances of tuberculosis, in descending order, were as follows: Areas of pulmonary consolidation consistent with pneumonia; nodular shadows consistent with pulmonary fibrosis; calcification, pleural effusions or lymph node enlargement; and cavity formation or miliary shadows (7). However, Chen *et al* found that the typical observations of tuberculosis in descending order of occurence were mediastinal lymphadenopathy, pleural effusion and fibrocalcific lesions on chest imaging (1). In a previous study of 1,161 patients admitted to hospital for tuberculosis, the cervical lymph nodes were most frequently involved (63.3%), followed by the mediastinal lymph nodes (26.7%) and axillary lymph nodes (8.3%) (8). Mediastinal lymphadenopathy may occur as a complication of pulmonary tuberculosis or as a primary disease without pulmonary involvement (9). Lymph node tuberculosis is an important issue in developed countries and must be considered in differential diagnosis. In addition, negative results for the identification of *Mycobacterium tuberculosis* in the lymph nodes does not exclude the diagnosis of lymph node tuberculosis (8). With the exception of the involvement of the lymph nodes or lungs, disseminated tuberculosis involves a number of other organs, which may be confused with *Candida* (10). In the present case, the CT initially presented mediastinal and hilar enlarged lymph nodes, followed by supraclavicular fossa lymphadenopathy and, thus, AML infiltration was suspected. However, several cycles of chemotherapy did not resolve the lymphadenopathy, even when the patient had achieved complete remission. AML infiltration was excluded, and nodular shadows and obstructive pneumonia appeared as the disease became aggravated in the chest CT. Therefore, it was necessary to differentiate between tuberculosis, fungal infection and lymphoma.

In a previous study by Chen *et al*, positive culture(s) of *Mycobacterium* from sputum and/or tissue were determined as the standard for establishing the diagnosis of tuberculosis in adults (1). However, a number of patients had no sputum or complained only of a dry cough when without extensive infiltration of the lung or pleural effusion, or developed extrapulmonary tuberculosis only. In addition, immunosuppressed patients are less likely to exhibit positive sputum staining for AFB (6,11). In the current case report, the patient had a prolonged dry cough and even when the patient coughed white frothy sputum, the sputum staining for AFB and sputum cultures for fungi and bacteria were all negative. Bronchoalveolar lavage is reported to be an effective technique for patients with hematological malignancies and pulmonary tuberculosis (12). Biopsy of the target lesion showing granulomas, positive AFB staining and culture for mycobacteria are essential to differentiate tuberculosis infection from other infections (11,13). Certain previous studies have considered polymerase chain reaction as one of the diagnostic methods for *M. tuberculosis* (3,10,11). In the present study, the pathology of biopsy specimens from the supraclavicular fossa lymph node and lung nodular by FB showed caseous necrosis, epithelioid cell nodules and multinuclear giant cells. However, the staining of biopsy specimens and bronchial lavage fluid were all negative.

Immunosuppressed patients are less likely to develop a positive tuberculin skin test (6). However, the current patient exhibited a marked response to the PPD skin test and PPD IgG Ab in the blood was also positive, which aided the diagnosis of tuberculosis. Among a number of patients with hematological malignancies, the patients developed tuberculosis during or following consolidation or maintenance chemotherapy (3,6,13). For tuberculosis with hematological disorders, the main predisposing factors were cytotoxic chemotherapy and steroid therapy, in descending order (7). However, in the current study, the onset of tuberculosis was prior to the diagnosis of AML and this disseminated during chemotherapy. The deterioration of tuberculosis may have been caused by the immunosuppressive therapy. However, the correlation between tuberculosis onset and AML remains unclear.

Good clinical response to tuberculosis treatment has been reported in adult patients with underlying hematological malignancies treated with isoniazid, rifampicin, pyrazinamide and ethambutol for two months followed by isoniazid and rifampicin for an additional four to 10 months (14). Occasionally, empirical antituberculosis therapy is necessary when the clinical and radiological features are markedly suggestive of tuberculosis, particularly in patients living in endemic areas (7). The response to antituberculosis treatment has been defined when patients become afebrile and the lesions subside, which may be considered as possible evidence for the establishment of tuberculosis (3). In the current case report, the patient was administered three antituberculosis drugs as diagnostic treatment prior to the definite diagnosis of tuberculosis. The patient became afebrile later, which increased the suspicion of tuberculosis. Due to the patient’s elevated liver enzymes, only two antituberculosis drugs were administered and tuberculostatic therapy was interrupted for a period of time. Additionally, the patient demonstrated poor compliance, which delayed diagnosis and aggravated the conditions. With the aid of the constitutive BM infiltration, radiographic examination, morphological observations of the two biopsies and the beneficial effect of antituberculosis drugs, the diagnosis of tuberculosis was finally determined and the patient’s symptoms and radiographic observations improved.

In conclusion, the current case report presents a case of scrofula and pulmonary tuberculosis developing with AML. It is important to distinguish tuberculosis in adults with AML from other causes of fever, mediastinal masses in radiographic observations and hemophagocytosis in the bone marrow. We highlight the importance of being aware that these conditions may coexist, and clinicians must be careful to identify the occurrence of new and rapidly progressive symptoms in patients with an established diagnosis, in case of adverse outcomes due to delayed diagnosis and treatment.
